# The Surgical Aspect of Hepatic Abscess

**Published:** 1890-02-22

**Authors:** 


### SURGERY.

### THE SURGICAL ASPECT OF HEPATIC ABSCESS.

Mr. Rickman J. Godlee, surgeon to University College Hospital, has given two lectures on the above subject. (*British Medical Journal.*) The author details various cases in which the symptoms led to exploration and puncture of the liver with varying results. Now exploration and puncture of the liver has been by some regarded as rather a trivial operation than otherwise. For example, Dr. George Harley has advocated it, not only for abscess but for the relief of an inflammatory condition, but Mr. Godlee utter a well-timed warning. Profuse bleeding may follow, especially in a person subject to bleeding from slight cause, or who has a scorbutic condition of system. A case of the kind is given. Mr. Godlee observes, "A puncture of the liver, if there be no adhesions, is never free from danger from this cause" (hæmorrhage). A large vessel wounded near the surface of the liver may in a healthy subject, give rise to fatal bleeding, "and thus, though considering the great rarity of the occurrence, we must not be deterred from doing it, I think it behoves us not to speak too lightly of the performance to the patient or his friends." Supposing pus to have been discovered, Mr. Godlee agrees with most recent authors that the best plan is to give exit to it at once by making a hole large enough to admit the finger, and afterward inserting a drainage tube of the same size. Most likely, if the abscess forms a prominent swelling, and almost certainly if the skin be reddened, the surface of the liver will be adherent to the abdominal walls. But it is not safe to assume the presence of these adhesions. Mr. Godlee therefore advises to cut down cautiously through the abdominal walls, and to ascertain the state of the case. If no adhesions be met with it is not difficult by means of a full-curved needle held in a needle-holder to pass a series of stitches through the parietal peritoneum, and pretty deeply into the substance of the liver. If the abcess is pointing in the side the incision should be below the normal line of the pleura, if possible, roughly not more than two inches above the margin of the ribs in the mid-axillary line. If the pleura be opened it will be necessary to stitch the diaphragm to the costal pleura. Now we doubt whether all this stitching is really essential. Mr. Godlee himself observes that he does not think the escape of a little pus into the peritoneum would of necessity prove fatal. As a matter of fact we believe it very often occurs, for surgeons have not been in the habit of making such stitches; and it is unfortunate that the one case given by Mr. Godlee where the liver was stitched terminated fatally. In making the first incision, if any considerable depth of liver tissue has to be traversed, very free hæmorrhage is likely to occur. This, says Mr. Godlee, must be treated by plugging the wound with the finger for a minute or two, when in all probability it will cease. But if this be not the case a plug of antiseptic material must be left in for a day or two. But the best way of opening an abscess in the liver is with a trocar and canula, and making the opening larger by passing in a pair of dressing forceps closed and drawing them out open. Mr. Godlee has had an instrument made for this purpose. It consists of a trocar which, as well as the corresponding canula, is grooved on one side, down which groove a fine pair of dressing forceps may be run, and brought out with expanded blades. In his second lecture Mr. Godlee details various cases, mentioning, very correctly, that it is difficult to meet with an instance which does not present some special peculiarity. Most of the cases appear to have been far advanced when seen, as indeed must be the state when a patient contracts the abscess in the East. One case is, however, related of liver abscess in a person who had never been out of England. The abscess was small, pointed at the epigastrum and was opened there. It was treated with carbolic acid gauze, and a good recovery ensued. In contradistinction to this, we may refer to Mr. Godlee's Case XV. An Anglo-Indian had previously suffered from enlargement of the liver. On June 17, 1889, he had a stitch in the right side, and friction was heard at the base of the lungs, followed by slight dullness. He came home on twelve months' leave. He was seen by Dr. Godlee in September, 1889, and was "then in fair general health, walking about, and feeding like anyone else." But he had a prominent red swelling over the right eighth cartilage. The diagnosis lay between tubercular disease of a costal cartilage and abscess of the liver. On incision, it was found to be abscess of the liver. The patient recovered. There are, however, other forms of deep-seated liver abscess more difficult to diagnose than even the above case. For these abscesses often do not come to the surface, and the diagnosis must be altogether based on constitutional symptoms, or on the discovery of pus by exploration of the liver. Notwithstanding what has been said by Dr. George Harley, we do not know enough of the results of exploration of the liver, neither do we know enough of the results of endeavouring to cure an abscess of the liver by aspiration. At present aspiration alone has very rarely ever cured a liver abscess; for after aspiration, in our experience, this close method has to be abandoned for the open method. A great deal has been written on the proper treatment of abscess of the liver, some advocating one method, others another. But success depends not so much on the method adopted as on the size and position of the abscess, and the constitution and strength of the patient.

